# Patterns of e-Cigarette Use and Smoking Cessation Outcomes: Secondary Analysis of a Large Randomised Controlled Trial to Inform Clinical Advice

**DOI:** 10.1093/ntr/ntaf240

**Published:** 2025-12-10

**Authors:** Francesca Pesola, Katie Myers Smith, Dunja Przulj, Daniella Ladmore, Anna Phillips-Waller, Hayden McRobbie, Peter Hajek

**Affiliations:** Wolfson Institute of Population Health, Queen Mary University of London, London, UK; Wolfson Institute of Population Health, Queen Mary University of London, London, UK; Wolfson Institute of Population Health, Queen Mary University of London, London, UK; Wolfson Institute of Population Health, Queen Mary University of London, London, UK; Wolfson Institute of Population Health, Queen Mary University of London, London, UK; Wolfson Institute of Population Health, Queen Mary University of London, London, UK; National Drug and Alcohol Research Centre, University of New South Wales, Sydney, Australia; Wolfson Institute of Population Health, Queen Mary University of London, London, UK

## Abstract

**Introduction:**

Limited data exist on how to use e-cigarettes (EC) to optimize smoking cessation.

**Methods:**

We examined associations between patterns of EC use and outcomes at 1 year in a large trial (*N* = 886) comparing nicotine replacement therapy (NRT) and EC.

**Results:**

Use of tobacco flavor was limited and associated with a lower smoking cessation rate compared to other flavors (relative risk; RR = 0.56, 95% CI = 0.35 to 0.89). EC users reduced nicotine strength over time. Abstainers using EC reported lower urges to smoke than abstainers using NRT at both weeks 1 (*b* = −0.25, 95% CI = −0.45 to −0.04) and 4 (*b* = −0.37, 95% CI = −0.58 to −0.16). Participants using both cigarettes and EC (dual users) at week 1 were more likely to stop smoking at week 4 than exclusive smokers (RR = 4.45, 95% CI = 1.96 to 10.10). Dual users at 4 weeks and 6 months were also more likely to achieve validated 50% reduction in smoke intake at 1 year (RR = 2.37, 95% CI = 1.36 to 4.11 and RR = 4.56, 95% CI = 2.71 to 7.66, respectively).

**Conclusions:**

Non-tobacco flavors were preferred and may be also more effective. Urges to smoke were lower in EC users than in users of NRT. Dual use was associated with a reduction in smoking and quitting smoking later on.

**Implications:**

Clinicians advising smokers wanting to use EC as a quitting aid can consider recommending non-tobacco flavors; explaining that EC reduce urges to smoke more than combination NRT; and reassuring those worried about dual use that such use is associated with reduced smoking and a higher chance of stopping smoking altogether later on.

## Introduction

There is good evidence that e-cigarettes (EC) are effective in helping smokers quit.^1^ Knowledge however remains limited regarding practical treatment details, eg, whether and how usage patterns and product characteristics affect outcomes.

In several reports, mostly analyzing the US Population Assessment of Tobacco and Health (PATH) data, using EC daily (as opposed to using EC infrequently) was associated with successful reduction or cessation of smoking.^2–6^ Daily use could be either a marker of success (smokers for whom EC “worked” were using them more frequently) or its precursor (EC “worked” because they were used daily). A UK survey with 1-year follow-up found daily vaping associated with a reduction in smoking, but not with smoking cessation.^7^ A case-controlled study reported that users of both cigarettes and EC (dual users) who later on stopped smoking altogether were more likely to use EC with higher nicotine delivery and be less concerned about health risks of vaping^8^ We are not aware of any evidence-based advice or guidance on how to use EC most effectively.

Participants in trials of EC for smoking cessation tended to prefer sweet flavors over tobacco and menthol.^9^ In one such trial, participants who switched to vaping fully were more likely to use fruit flavors than dual users; and at 6 months, they used e-liquids with a lower nicotine strength than dual users (6.3 vs. 8.2 mg/mL).^10^

Over 1 year, 35.0% of participants did not change device type, nicotine concentration, or flavor type. However, 35% of participants changed device type, 31.3% changed nicotine concentration, and 46.3% changed flavor at least once. The most common change in device type was switching from open to closed system devices (67.9%). The rate of switching from low to high nicotine (≤24 vs. >24 mg/mL) was more common than the reverse (56.6% vs. 43.4%). The most common change in flavor was switching between Fruit/Sweet and Hybrid (Fruit/Sweet with a cooling additive, 75.7% of flavor switching events).

We conducted a secondary analysis of data from a randomized controlled trial comparing EC and nicotine replacement therapy (NRT)^11^ that can provide some practical information for smokers and clinicians advising them. The main study report focused on the comparison of the randomized groups to assess treatment safety and efficacy. Here we look at outcomes in participants who used, or did not use, EC regularly during their quit attempt to examine the issues listed below.

### How Does EC Use Change Over Time, and Are Different Use Characteristics Associated with Smoking Status?

It is not clear whether smokers should be advised to stick to the EC product they start with (particularly where this is provided by stop-smoking services, as is the case in the UK), or whether they should be encouraged to find themselves a product, flavor, and nicotine strength that they prefer. Data on what proportion of smokers provided with a starter pack migrate to other products and whether product changes are associated with success in stopping smoking could guide relevant recommendations. Several jurisdictions are also currently considering banning EC flavors other than tobacco to make vaping less attractive to young people. Trial data can provide new information on flavor preferences among smokers using EC to stop smoking, and associations between flavors and smoking status.

### Are People Who Do Not Manage to Stop Smoking Early on and Also Use EC (Dual Users) More or Less Likely to Stop Smoking Altogether Than Those Who Do Not Use EC?

Smokers attempting to quit with the help of EC are typically encouraged to set a target quit date (TQD) and stop smoking altogether early on, as with other stop-smoking approaches. Some, however, continue to smoke and use their EC; and this is more common than with NRT.^11–13^ There are concerns that dual use may undermine stopping smoking later on.^14^ Conversely, obtaining nicotine from an alternative source could reduce the reward from smoking and facilitate quitting.^15^ Trial data allow comparisons of trajectories of participants who did not manage to stop smoking early on and either did or did not use EC. The results can provide a basis for several advice scenarios: warning people who smoke that dual use may undermine their chances of successfully stopping smoking; reassuring them that dual use is associated with stopping smoking altogether; or advising that such use does not affect future smoking cessation.

### Does EC Use Promote or Impede Reduction in Smoking?

There are concerns that dual use may increase exposure to toxic chemicals,^16^ but it is also possible that receiving nicotine from another source reduces smoking and thus decreases such exposure.^17^ Trial data include information on the association between dual use and smoke intake, verified objectively by a carbon monoxide (CO) reading.

### How Should Clinicians Explain the Effect of ECs on Stopping Smoking?

With all licensed stop-smoking medications, clinicians typically explain to smokers that the medication facilitates smoking cessation by reducing cigarette withdrawal discomfort, particularly urges to smoke. EC users abstaining from smoking reported lower withdrawal discomfort and urges to smoke at 6 weeks than participants who continued to smoke, but without a comparison with abstainers not using EC, the finding is difficult to interpret.^18^ Effects of EC on urges to smoke have been demonstrated primarily over short time periods.^19–21^ The Trial of Electronic Cigarettes (TEC) trial data allow exploration of whether this also applies across the full period of acute withdrawal discomfort that in smokers seeking treatment typically spans the first 4 weeks of abstinence from smoking.^22^

## Materials and Methods

### Design

The parent trial for this secondary analysis was TEC, conducted from May 2015 through February 2018. For details, see^11^ (registration ISRCTN60477608). In brief, the trial included 886 smokers accessing stop-smoking services and not currently using NRT or EC. They were randomized to receive a combination of NRT products of their choice (typically a nicotine patch plus a shorter acting NRT such as a nicotine inhalator or mouth spray) supplied for up to 3 months; or an EC starter pack comprising of a refillable EC device (The UK Ecig Store OneKit) and one 30 mL bottle of 18 mg/mL nicotine e-liquid (expected to last for about 2 weeks) with instructions to buy further supplies of e-liquids with strength and flavors of their choice themselves. One-year sustained biochemically validated abstinence from smoking rates were 18.0% in the EC group and 9.9% in the NRT group, with EC being also more cost-effective than NRT.^23^

The aims of the present report were to:

1. Examine patterns of EC use during the 1-year study period and their associations with abstinence from smoking.
2. Determine if EC use in people unable to stop smoking early on predicts smoking status later.
3. Explore links between EC use in non-abstainers and exposure to cigarette smoke.
4. Examine the association between EC use and urges to smoke at 1 and 4 weeks.

### Measures

Smoking status was collected at 1 week, 4 weeks, 6 months, and 1 year. Expired air CO readings were taken at baseline, 4 weeks and 1 year when they were used to validate self-reported abstinence from smoking. Abstinence from smoking at week 1 was defined as reporting not a puff in the previous 7 days. Abstinence at week 4 was defined as reporting not a puff in the previous seven days validated by a CO reading of <8 parts per million (ppm).

Abstinence from smoking at 1 year was defined as per primary outcome in the parent trial, ie, smoking no more than 5 cigarettes from 2 weeks after the TQD with no smoking in the past 7 days, confirmed by a CO reading of <8 ppm. Participants lost to follow-up were classified as non-abstainers, as per Russell Standard.^24^

Urges to smoke were assessed via Mood and Physical Symptoms Scale,^25^ which was administered at baseline and at weeks 1 and 4, with possible scores ranging from 1 to 6.

Among participants who did not manage to stop smoking, those who reduced both their self-reported cigarette consumption and their CO reading by at least 50% were classified as “reducers.”

Regarding product use, at each time point, participants who reported using EC on at least 1 day per week were classified as current users. Participants reporting smoking and using EC were classified as dual users. Participants who used both NRT and EC concurrently (a small number at any time point) were included as EC users in the primary analyses and excluded in sensitivity analyses. Participants using EC were asked to provide information on the strength and flavor of their e-liquids at each timepoint. Regarding EC devices, we differentiate between “refillable EC” with a tank that users fill with e-liquid from separate e-liquid bottles (this was provided to the EC arm in the parent study); “cartridge-based EC which use replaceable pre-filled cartridges containing e-liquid; and disposable EC” which are discarded whole when empty.

### Statistical Methods

To explore patterns of EC use in the EC arm, we report: the number (%) of participants who switched from the EC provided (OneKit) to another EC product, those who changed nicotine strength (from 1.8% provided) and those who switched flavor (from tobacco provided); and the number (%) who reported using nicotine-free vapes. We also examined changes in nicotine strength of e-liquid, e-liquid flavors, and EC device among respondents using EC at 4 weeks and 1 year and estimated median change differences and their associated 95% CIs.

To examine whether continuing to use the original EC product versus changing to a product of clients’ choice and using tobacco versus non-tobacco flavored e-liquids was associated with abstinence from smoking and smoking reduction at 1 year, we used generalized linear model (GLM) with family set as binomial with a log link to estimate risk ratios. If the model failed to converge, we specified the family as Poisson with log link and robust standard errors.

To explore effects of EC use on other variables, we included all EC users, regardless of arm allocation. To assess if, among non-abstainers at earlier time points, nicotine product use was associated with abstinence at a later follow-up, we used the same GLM approach. A GLM was also used to assess links between nicotine products use and relapse rates and between products use and validated reduction in smoking at 1 year.

To explore the associations between nicotine product use and urges to smoke, we used linear regression models with urge scores treated as the outcome. All regression models were adjusted for sex, cigarette dependence (Fagerstrom Test for Cigarette Dependence; FTCD)^26^; scores and age as these baseline characteristics were associated with abstinence in this sample. All analyses were also adjusted for study center. As in the main analyses of the trial, participants who died (one in each intervention arm) were excluded from the analyses, reducing the total *N* from 886 to 884.

The section on changes in EC use over time concerns just the EC arm of the trial, because the EC arm was started on EC. All other sections include EC users from both study arms.

The analyses are exploratory and were conducted using Stata v18.0.^27^

## Results

### Patterns of EC Use Over Time and Smoking Status

Table 1 shows EC use over time and changes in device, e-liquid nicotine strength, and flavors among EC arm participants.

**Table 1:** EC Devices and E-Liquids used at 4 Weeks and at 1 Year in Participants Allocated to the EC Arm (*N* = 438)

	Use at 4 weeks	Use at 1 year
Using any EC, *N* (% of participants)	343 (78.3%)	235 (53.6%)
Using OneKit, *N* (% of EC users)	312 (91.0%)	88 (37.4%)
EC flavors in those providing the information^*^, *N* (%)		
	4 weeks, *N* = 343	1 year, *N* = 235
Still using tobacco flavor	193 (50.1)	104 (25.9)
Menthol	44 (11.4)	54 (13.5)
Fruit	87 (22.6)	133 (33.2)
Sweet flavors	24 (6.2)	53 (13.2)
Other^	37 (9.6)	57 (14.2)
Nicotine content of e-liquid in those providing the information, *N* (%)		
	Week 4 (*N* = 343)	1 year (*N* = 235)
>1.8%	8 (2.3)	5 (2.1)
1.8%	238 (69.4)	66 (28.1)
1%–1.7%	69 (20.1)	56 (23.8)
0.1%–0.9%	24 (7.0)	85 (36.2)
0%	4 (1.2)	23 (9.8)
Change in nicotine strength from 4 weeks to 1 year		Median paired difference = 0.6%, 95% CI = 0.4–0.6

CI = confidence interval; EC = e-cigarettes.

^*^Some participants reported more than one flavor.

^ Includes answers such as “don’t know” and “unsure.”

Just over a third of participants using EC at 1 year were still using the original device, the rest had purchased new devices of their own choice. Almost all were still using refillable products (226 out of 235, 96.2%), with only 8 (5.4%) using pods,^11^ heated tobacco product (*N* = 1) or cartridge EC (*N* = 1) and 1 not providing this information.

Tobacco flavored e-liquids were unpopular. Half of EC users switched to other flavors within the first 4 weeks and two thirds abandoned them by 1 year. Fruit flavors were the most popular (Table 1). Quit rates were lower among participants still using tobacco flavored e-liquid at 1 year than in those who switched to other flavors (20.2% vs. 36.9%, relative risk [RR] = 0.56, 95% CI = 0.35 to 0.89). Reduction in smoking at 1 year was similar among non-abstainers using tobacco and non-tobacco flavors (27.62% vs. 27.8%, respectively; RR = 1.03, 95% CI = 0.63 to 1.70).

Participants who provided information about the nicotine content of their e-liquid both at 4 weeks and at 1 year (*N* = 214) reduced the nicotine strength of their e-liquid (median = 1.8%; IQR = 1.6–1.8 at 4 weeks vs. median = 1.0%, IQR = 0.5–1.8 at 1 year, median paired difference = 0.6%, 95% CI = 0.4–0.6). About 10% of participants using EC at 1 year and providing relevant data were using nicotine-free e-liquid (12 [16.9%] among 1-year abstainers).

Among participants who provided information on nicotine content of their e-liquid at 4 weeks (*N* = 343), liquid strength at 4 weeks was not related to abstinence or CO-validated reduction in smoking at 1 year (RR = 0.99, 95% CI = 0.95–1.04 and RR = 0.98, 95% CI = 0.93–1.04, respectively), but most participants (69%) were using the same strength at 4 weeks (1.8%).

At 1 year, among participants still using EC, abstainers were using lower strength EC e-liquids than non-abstainers (median = 0.6% [IQR: 0.3–1.5], *n* = 71 vs. 1.2% IQR: 0.6–1.8, *N* = 161, Mann–Whitney *z* = 3.0, *p* = .002).

### EC Use and Subsequent Smoking Cessation

Baseline characteristics were comparable between participants who did and did not use EC (see Supplementary Table S1). Those still smoking and using EC (dual users) at earlier time points were more likely to be abstinent from smoking at later time points than those who were only smoking (Table 2). Excluding participants using both EC and NRT (*N* = 9 at week 1 and *N* = 8 at week 4) did not change the results (see Supplementary Table S2).

**Table 2:** Dual use and Smoking Status at the Next Follow-Up

	*N* (%) abstinent from smoking among participants using vs. not EC at previous time point	
	Week 4^**^	1 year^**^
EC use at week 1 (*N* = 197 dual users and *N* = 121 exclusive smokers)	47 (23.9%) vs. 6 (5.0%) RR = 4.45 (1.96–10.10)	17 (8.6%) vs. 1 (0.8%)
EC use at week 4 (*N* = 161 dual users and *N* = 239 exclusive smokers)		5 (3.1%) vs. 1 (0.4%)

RR = relative risk; CI = confidence interval; EC = e-cigarettes.

^**^With *N* = 1 in non-user groups, RR (95% CIs) are unstable and hence not calculated.

Among 121 abstainers at week 1 and 239 at week 4 classified as not using nicotine products, 10 (8.3%) and 30 (12.6%) were so classified because they did not provide any information on product use.

### EC Use in Non-Abstainers and Consecutive Validated Reduction in Smoking

Compared to participants who only smoked, dual users at 4 weeks were more likely to achieve validated reduction in smoke intake of at least 50% at 1 year (*N* = 30, 19.2% vs. *N* = 18, 7.6%, RR = 2.37, 95% CI = 1.36–4.11). Excluding participants who reported smoking and using both EC and NRT did not change the results (RR = 2.39, 95% CI = 1.37–4.16).

Compared to participants who only smoked, dual users at 6 months were more likely to achieve smoking reduction at 1 year (*N* = 50, 24.6% vs. *N* = 18, 5.2%, RR = 4.56, 95% CI = 2.71–7.66). Excluding participants who reported smoking and using both NRT and EC did not change the results (RR = 4.87, 95% CI = 2.87–8.29 for EC).

### EC Use and Urges to Smoke

Only one abstainer at week 1 and eight at week 4 were not using any nicotine products. In the overall sample, participants using EC had lower urges to smoke (*N* = 390, mean = 2.9, SD = 1.0) than those using NRT (*N* = 363, mean = 3.2, SD = 0.9; adjusting for age, sex and FTCD: *b* = −0.28, 95% CI = −0.42 to −0.15). Abstainers using EC reported lower urges to smoke (*N* = 194, mean = 2.7, SD = 1.01) than those using NRT (*N* = 152, mean = 2.9, SD = 0.89; adjusting for age, sex, and FTCD: *b* = −0.25, 95% CI = −0.45 to −0.04). Excluding three participants who were concurrently using EC and NRT did not change the results (whole sample: *b* = −0.29, 95% CI: −0.42 to −0.15; abstainers: *b* = −0.25, 95% CI = −0.45 to −0.05).

Participants using EC had lower urges to smoke (*N* = 357, mean = 2.4, SD = 1.1) than those using NRT (*N* = 279, Mean = 2.7, SD = 1.0; adjusting for age, sex, and FTND: *b* = −0.33, 95% CI = −0.49 to −0.16). Abstainers using EC continued to report lower urges to smoke (*N* = 196, mean = 2.0, SD = 0.94) than those using NRT (*N* = 121, mean = 2.3, SD = 0.84; adjusting the model for age, sex, and FTCD: *b* = −0.37, 95% CI = −0.58 to −0.16). Excluding five participants who were concurrently using EC and NRT did not change the results (whole sample: *b* = −0.33, 95% CI = −0.50 to −0.16; abstainers: *b* = −0.39, 95% CI = −0.60 to −0.18).

## Discussion

Several of these results could be helpful to people who smoke and are trying to stop with the help of EC, and to clinicians trying to optimize smoking cessation outcomes.

Regarding EC devices, practically all regular vapers persisted in using refillable EC. This tallies with population data from England from the time of the trial.^28^ The trial preceded the more recent surge in popularity of disposable EC devices but this has been observed primarily among novice users.^29^ Refillable devices are cheaper over longer term but may be also preferred by people trying to stop smoking in markets such as the European Union and the UK because these countries limit nicotine content to 2% and cartridge-based devices with smaller batteries provide only low nicotine levels.^30^ This may not apply to countries like the United States where higher nicotine strength e-liquids are available.^31^

Regarding EC flavors, most studies provided e-liquids with tobacco flavors, assuming that this is more familiar to smokers than other flavors and so likely to be preferred. In this study, half of EC users opted for other flavors, mostly fruit, within the first 4 weeks and only a quarter of long-term EC users continued to use tobacco flavor at 1 year. This tallies with other studies reporting that smokers who successfully switch to vaping prefer non-tobacco flavors.^32^^,^^33^ Switching to non-tobacco flavor was also associated with a better 1-year outcome. Tobacco flavor may be more conducive to relapse (due to eg, association with smoking), or just less attractive and so less helpful than other flavors.

Regarding nicotine strength, participants started on 1.8% e-liquid and this remained the most popular choice at 4 weeks, when most participants were already buying their own e-liquid and had an opportunity to change their nicotine strength. This suggests that the current practice of recommending that smokers unable to quit unaided start with the highest nicotine strength allowed in the EU seems reasonable. During the year, most EC users reduced the nicotine strength of their e-liquid as has also been reported in previous studies.^34^ At 1 year, 10% of participants using EC were using nicotine-free e-liquid. Self-administration of nicotine on its own seems to be less reinforcing than when nicotine is combined with other chemicals in tobacco smoke.^35–37^ Some people who smoke may benefit from being informed that EC can provide an avenue to gradually wean themselves off nicotine.

Another finding of practical importance is that dual use was associated with stopping smoking later and in those who did not manage to stop smoking, it was linked with a substantial reduction in smoking. This was not just a reduction in cigarette consumption, which on its own may not convey much health benefit if the remaining cigarettes are smoked with more intensity,^38^^,^^39^ but an objectively validated reduction in smoke intake which would be accompanied by a reduction in health risks.^40^^,^^41^

The study data also provide an explanation of this. There is good evidence that NRT use reduces urges to smoke.^42^ In this study, participants using EC had even lower urges to smoke than those using NRT, throughout the initial 4 weeks of abstinence. This tallies with the finding of lower urges to smoke in the EC arm in the parent trial (3).

The main limitation of this study is its observational nature. EC use was self-selected. We controlled for the variables that were associated with abstinence (sex, cigarette dependence, and age) but there may have been other variables responsible for individual differences in EC use status that we did not measure, such as motivation to quit, and that could have contributed to at least some of the associations reported here. Further randomized studies are needed to test whether the links are causal. There were too few smokers at 6 months who managed to quit at 1 year (5 who used EC and 1 who did not) to allow robust statistical evaluation.

In summary, the current analyses have generated several findings that have implications for clinical practice. People who smoke and are trying to quit with the help of EC can be advised that EC use is associated with reduced urges to smoke, that those starting with high nicotine strength typically reduce the strength after successfully stopping smoking, and that most prefer non-tobacco flavors. Those unable to stop smoking altogether early on can be encouraged not to give up on EC use as such use is associated with a reduction in smoking and with stopping smoking altogether later.

## Supplementary Material

R2_Supplement_ntaf240

## Data Availability

The data that support the findings of this study are available from the corresponding author upon reasonable request.
