# Supplementary material for: Patterns of e-Cigarette Use and Smoking Cessation Outcomes: Secondary Analysis of a Large Randomised Controlled Trial to Inform Clinical Advice
Source: Nicotine Tob Res. 2025 Dec 10;28(6):1039–44. doi: 10.1093/ntr/ntaf240 (PMC13196703; doi:10.1093/ntr/ntaf240)
Supplement: R2_Supplement_ntaf240 [file r2_supplement_ntaf240.docx]

**Supplementary Table**

Pesola F, Myers-Smith K, Przulj D et al. E-cigarettes as a stop smoking aid: Secondary analysis of a large RCT to inform clinical advice

**eTable1. Baseline characteristics broken down by EC use (yes vs. no) at week 1.**

|  | **No EC use (N=493)** | **EC use**  **(N=391)** | **Chi-square** |
| --- | --- | --- | --- |
| **Female n(%)** | 237 (48.1) | 187 (47.8) | Chi^2^(1)=0.01, p=0.94 |
| **Employed n(%)** | 349 (70.8) | 266 (68.0) | Chi^2^(1)=0.78, p=0.38 |
| **Free prescription n(%)** | 198 (40.2) | 162 (41.4) | Chi^2^(1)=0.15, p=0.70 |
| **White** | 351 (72.1) | 282 (73.1) | Chi^2^(1)=0.10, p=0.75 |
| **Married** | 118 (23.9) | 103 (26.3) | Chi^2^(1)0.67, p = 0.41 |
| **Education**  Primary school  Secondary school  Further education  Higher education | 22 (4.5)  151 (30.6)  142 (28.8)  178 (36.1) | 19 (4.9)  120 (30.7)  102 (26.1)  150 (38.4) | Chi^2^(3)=0.96, p=0.81 |
| **Past NRT use** | 377 (76.5) | 285 (72.9) | Chi^2^(1)=1.49, p=0.22 |
| **Past EC use** | 200 (40.6) | 167 (42.7) | Chi^2^(1)=0.52, p=0.41 |
|  |  |  | **Mann-Whitney/t-test** |
| **Age median (IQR)** | 41 (33-51) | 41 (32-53) | z = 0.01, p=0.99 |
| **CPD median (IQR)** | 15 (10-20) | 15 (10-20) | z = 1.01, p=0.31 |
| **FTCD mean (sd)** | 4.6 (2.4) | 4.5 (2.4) | t(882)=0.65, p=0.52 |

**eTable 2:** Dual use and smoking status at the next follow-up. Sensitivity analysis with users of both EC and NRT excluded.

|  | N (%) abstinent from smoking among participants using vs not using EC at previous time point | |
| --- | --- | --- |
|  | Week 4 * | 1 year ** |
| EC use at week 1  (N=191 dual users and 121 exclusive smokers) | 45 (23.6) vs 6 (5.0)  4.42 (1.94-10.06) | 16 (8.4) vs 1 (0.8)  n/c |
| EC use at week 4  (N=153 dual users and N=239 exclusive smokers) |  | 5 (3.3) vs 1 (0.4)  n/c |

* RR (95%CI)

** With N=1 in non-user groups, RR (95% CIs) are unstable and hence not calculated
